# Proteasome inhibition suppresses the induction of lipocalin-2 upon systemic lipopolysaccharide challenge in mice

**DOI:** 10.1186/s13041-024-01147-w

**Published:** 2024-10-03

**Authors:** Jin-Sil Bae, Ji-Eun Heo, Kwon-Yul Ryu

**Affiliations:** Department of Life Science, University of Seoul, 163 Seoulsiripdae-ro, Dongdaemun-gu, Seoul, 02504 Republic of Korea

**Keywords:** Lipocalin-2, Lipopolysaccharide, Bortezomib, Astrocyte, Neuroinflammation

## Abstract

Lipocalin-2 (Lcn2), a protein secreted by immune-activated cells, including reactive astrocytes, is detrimental to the brain and induces neurodegeneration. We previously showed that Lcn2 levels are reduced in primary mouse astrocytes after treatment with the proteasome inhibitor bortezomib (BTZ). However, it remains unknown whether a decrease in Lcn2 levels after BTZ treatment can also be observed in vivo and whether it reduces neurotoxicity during lipopolysaccharide (LPS)-induced systemic inflammation in vivo. To answer these questions, we performed LPS challenge experiments by intraperitoneal injection in mice and found that Lcn2 levels were significantly increased in the brain, recapitulating in vitro experiments using astrocytes. Co-administration of LPS and BTZ reduced the Lcn2 levels compared to the levels in LPS-treated controls. Upon LPS challenge, the expression levels of glial marker genes were upregulated in the mouse brain. Of note, this upregulation was hampered by the co-administration of BTZ. Taken together, our results suggested that BTZ can reduce LPS-induced Lcn2 levels and may alleviate LPS-induced neuroinflammation and neurotoxicity in mice.

**Supplementary Information:**

The online version contains supplementary material available at 10.1186/s13041-024-01147-w.

Neuroinflammation is associated with aging and neurodegenerative diseases [1]. Hyperactivated neuroinflammation is harmful and can cause synaptic loss [2]. Neuroinflammation is believed to begin with microglia [3]. Secreted factors, such as cytokines, from activated microglia induce astrocyte activation [4]. Activated astrocytes can affect neuronal fate [5]. Although there are some disputes, activated astrocytes can be classified into A1 and A2 subtypes, according to traditional dichotomous concepts [6–9]. Pro-inflammatory cytokines activate astrocytes to the A1 subtype, secreting neurotoxins, such as lipocalin-2 (Lcn2). Anti-inflammatory cytokines activate astrocytes to the A2 subtype, secreting neurotrophic factors, such as brain-derived neurotrophic factor. These activated astrocytes have different transcriptome profiles. A1 astrocytes are neurotoxic, while A2 astrocytes are neuroprotective [7]. In this study, we used lipopolysaccharide (LPS) to recapitulate the neuroinflammation observed in neurodegenerative diseases. In mice, peripheral LPS challenge by intraperitoneal injection induces inflammation in the central nervous system, likely because the activated immune response in the blood circulates and stimulates microglia near the blood-brain barrier [10]. We demonstrated that an acute LPS challenge was sufficient to induce neuroinflammation in mice.

Lcn2, originally identified as a secretory protein in neutrophils, protects the innate immune system against bacterial infections by limiting bacterial growth and is involved in various pathophysiological processes [11]. In the brain, Lcn2 is involved the innate immune system. Under neuroinflammatory conditions, activated microglia and astrocytes change their characteristics and secrete toxic proteins, such as Lcn2, related to reactive gliosis. Previous studies showed that Lcn2 secreted from activated astrocytes is neurotoxic, and reactive gliosis is delayed in *Lcn2*-knockout (KO) mice under neuroinflammatory conditions [10, 12, 13]. Thus, Lcn2 is a key molecule involved in the progression of reactive gliosis. We recently demonstrated that LPS treatment of mouse primary astrocytes induced *Lcn2* expression [14]. However, *Lcn2* expression was reduced in the presence of bortezomib (BTZ), the first clinically approved proteasome inhibitor for multiple myeloma patients [15]. However, it is unclear whether BTZ can prevent or treat neuroinflammatory conditions in vivo.

In this study, we systemically challenged mice with an intraperitoneal injection of LPS and investigated whether Lcn2 levels increased in vivo. Under normal conditions, Lcn2 could not be detected in the cortical brain regions by immunoblot analysis, suggesting very low basal levels (Fig. 1A). However, LPS administration dramatically increased Lcn2 production, which was also observed in the serum, indicating that it may have spread body-wide through the bloodstream (Fig. 1B). Next, we investigated whether proteasome inhibition using BTZ could reduce Lcn2 production in vivo, as observed in primary astrocytes. We treated the mice with LPS alone or with LPS and BTZ simultaneously via intraperitoneal injection. After 24 h of LPS treatment, Lcn2 levels were dramatically increased in the brain (Fig. 1C). Increased Lcn2 levels were not observed when mice were co-treated with LPS and BTZ. Serum Lcn2 levels also increased after systemic LPS challenge and were lower when co-treated with LPS and BTZ (Fig. 1D). Therefore, the effect of BTZ on Lcn2 after LPS treatment was also observed in mice. To mimic neuroinflammatory stress prior to BTZ administration, mice were challenged with LPS and then treated with BTZ later. Mice were challenged with LPS for 24 h and treated with BTZ at 6, 12, or 18 h after the start of the LPS challenge (Fig. 1E). Early and longer BTZ treatment was more effective at reducing brain Lcn2 levels during LPS challenge (Fig. 1F). Lcn2 is known to be degraded quickly by the autophagic pathway, with a half-life of approximately 30 min in cultured primary astrocytes [14]. Therefore, inhibiting *de novo* Lcn2 production by BTZ can reduce Lcn2 levels in cells. Similarly, BTZ treatment also reduced Lcn2 levels, which were already increased in LPS-challenged mice.

**Fig. 1:**
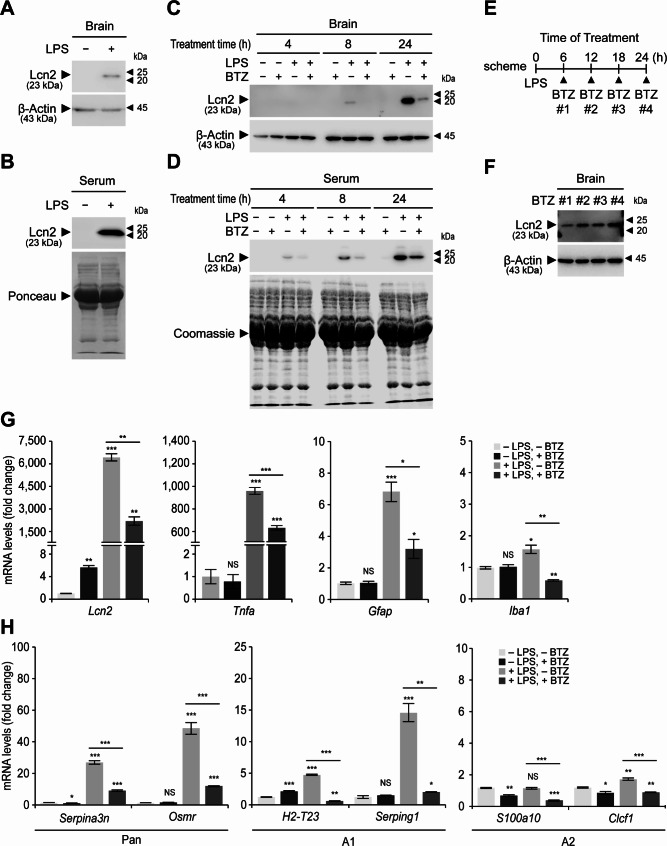
Bortezomib co-treatment inhibited Lcn2 production and upregulation of *Lcn2* during systemic LPS challenge in mice. (**A**) Immunoblotting detection of Lcn2 was performed using mouse brain lysates (cortical region). Ten-week-old mice were treated with lipopolysaccharide (LPS; 0 or 5 mg/kg body weight) by intraperitoneal injection. One day later, mice were sacrificed, and brains were dissected. (**B**) Lcn2 detection in mouse serum was performed by immunoblotting after 1 d of LPS treatment (0 or 5 mg/kg). (**C, D**) Immunoblotting detection of Lcn2 in the mouse brain (cortical region) and serum was performed after treatment with LPS (0 or 5 mg/kg) and/or bortezomib (BTZ; 0 or 2 mg/kg) for 4, 8, or 24 h. (**E**) Schematic representation of experiments. The arrowheads indicate the delayed time points when BTZ treatment began. ‘BTZ #1’, ‘BTZ #2’, and ‘BTZ #3’ refer to BTZ treatment for the last 18, 12, or 6 h, respectively, during the 24-h LPS treatment. ‘BTZ #4’ indicates that BTZ was not administered during the 24-h LPS treatment. (**F**) Lcn2 detection in the mouse brain (cortical region) was performed after LPS (0 or 5 mg/kg) and/or BTZ (0 or 2 mg/kg) treatment according to the scheme shown in (**E**). (**G, H**) Ten-week-old mice were treated with LPS (0 or 5 mg/kg) and/or BTZ (0 or 2 mg/kg) by intraperitoneal injection. One day later, brains (cortical region) were dissected for RNA isolation and subjected to qRT-PCR analysis. The expression levels of *Lcn2*, *Tnfa*, *Gfap*, *Iba1*, pan-astrocyte markers (*Serpina3n*, *Osmr*), A1 astrocyte markers (*H2-T23*, *Serping1*), and A2 astrocyte markers (*S100a10*, *Clcf1*) were determined, normalized against *Gapdh* levels, and expressed as the fold change relative to the control (− LPS, −BTZ; *n* = 3). Data are expressed as means ± standard error of the mean (SEM) from the indicated number of samples. *p < 0.05, **p < 0.01; ***p < 0.001 vs. the negative control (− LPS, −BTZ) or between two groups as indicated by the horizontal bars. NS, not significant. For immunoblotting analysis, β-actin (β-Actin), Ponceau staining, or Coomassie blue staining was used as a loading control. Representative images of immunoblots are shown from duplicate experiments

To determine whether low Lcn2 levels in the brain after LPS and BTZ co-treatment were due to reduced *Lcn2* expression, we determined the mRNA levels using qRT-PCR analysis. *Lcn2* expression was markedly upregulated after LPS administration, but not after LPS and BTZ co-treatment (Fig. 1G). Co-treatment with LPS and BTZ also downregulated *Tnfa*, *Gfap*, and *Iba1* expression levels, all significantly increased after LPS exposure. Therefore, proteasome inhibition not only reduced the expression of neurotoxin Lcn2, but also reduced the expression of proinflammatory cytokine Tnf-α, astrogliosis marker Gfap, and microglia activation marker Iba1. LPS challenge increases pan and A1 marker expression, but not A2 marker expression in cultured primary astrocytes [4]. In the mouse brain, we also observed increased pan and A1 marker expression after LPS administration, which were markedly downregulated by LPS and BTZ co-treatment (Fig. 1G). As observed in cultured primary astrocytes, A2 marker expression in the mouse brain was not altered by LPS administration. LPS and BTZ co-treatment had a smaller effect on A2 marker expression than pan and A1 marker expression. Taken together, BTZ co-treatment prevented reactive gliosis toward the A1 subtype and neuroinflammation in the mouse brain during systemic LPS challenge.

Our acute model of LPS challenge was sufficient to activate astrocytes to the A1 subtype and induce neuroinflammation in mice. In this study, we demonstrated that BTZ effectively reduced neurotoxic Lcn2 levels in the mouse brain. As BTZ does not cross the blood-brain barrier (BBB), it may not directly affect the brain. However, by reducing systemic inflammatory responses in the systemic LPS challenge model, it seems to indirectly decrease brain Lcn2 levels and neuroinflammation. Thus, our systemic approach is effective for modulating neuroinflammation through peripheral interventions. In fact, LPS is known to damage or disrupt the BBB, which may allow BTZ to penetrate the barrier. It is important to note that, while BTZ may not cross the BBB during mild immune responses, it could penetrate the BBB during severe inflammation or when the BBB is compromised. Thus, during LPS challenge, we cannot exclude the possibility that it may exert direct effects as well. Nonetheless, we suggest that Lcn2 is a potential therapeutic target that can be downregulated by BTZ under neuroinflammatory conditions, which is a novel strategy to effectively reduce Lcn2 levels and alleviate neuroinflammation. In summary, our study demonstrated that BTZ administration in LPS-challenged mice may help reduce the neurotoxicity induced by Lcn2 secreted from reactive astrocytes. This treatment strategy can be applied to various neurodegenerative diseases associated with neuroinflammation.

## Electronic supplementary material

Below is the link to the electronic supplementary material.

Supplementary Material 1

## Data Availability

All data analyzed in this study were included in this article. Materials and methods are presented in the supplementary information.

## Abbreviations

Lcn2: Lipocalin-2
LPS: Lipopolysaccharide
BTZ: Bortezomib

## References

[1] Zhang W, Xiao D, Mao Q, Xia H. Role of neuroinflammation in neurodegeneration development. Signal Transduct Target Ther. 2023;8(1):267. 37433768 10.1038/s41392-023-01486-5 PMC10336149

[2] Rao JS, Kellom M, Kim HW, Rapoport SI, Reese EA. Neuroinflammation and synaptic loss. Neurochem Res. 2012;37(5):903–10. 22311128 10.1007/s11064-012-0708-2 PMC3478877

[3] Leng F, Edison P. Neuroinflammation and microglial activation in Alzheimer disease: where do we go from here? Nat Rev Neurol. 2021;17(3):157–72. 33318676 10.1038/s41582-020-00435-y

[4] Liddelow SA, Guttenplan KA, Clarke LE, Bennett FC, Bohlen CJ, Schirmer L, et al. Neurotoxic reactive astrocytes are induced by activated microglia. Nature. 2017;541(7638):481–7. 28099414 10.1038/nature21029 PMC5404890

[5] Lawrence JM, Schardien K, Wigdahl B, Nonnemacher MR. Roles of neuropathology-associated reactive astrocytes: a systematic review. Acta Neuropathol Commun. 2023;11(1):42. 36915214 10.1186/s40478-023-01526-9 PMC10009953

[6] Escartin C, Galea E, Lakatos A, O’Callaghan JP, Petzold GC, Serrano-Pozo A, et al. Reactive astrocyte nomenclature, definitions, and future directions. Nat Neurosci. 2021;24(3):312–25. 33589835 10.1038/s41593-020-00783-4 PMC8007081

[7] Li K, Li J, Zheng J, Qin S. Reactive astrocytes in neurodegenerative diseases. Aging Dis. 2019;10(3):664–75. 31165009 10.14336/AD.2018.0720 PMC6538217

[8] Liddelow SA, Barres BA. Reactive astrocytes: production, function, and therapeutic potential. Immunity. 2017;46(6):957–67. 28636962 10.1016/j.immuni.2017.06.006

[9] Sofroniew MV. Astrocyte reactivity: subtypes, States, and functions in CNS innate immunity. Trends Immunol. 2020;41(9):758–70. 32819810 10.1016/j.it.2020.07.004 PMC7484257

[10] Qin L, Wu X, Block ML, Liu Y, Breese GR, Hong JS, et al. Systemic LPS causes chronic neuroinflammation and progressive neurodegeneration. Glia. 2007;55(5):453–62. 17203472 10.1002/glia.20467 PMC2871685

[11] Flo TH, Smith KD, Sato S, Rodriguez DJ, Holmes MA, Strong RK, et al. Lipocalin 2 mediates an innate immune response to bacterial infection by sequestrating iron. Nature. 2004;432(7019):917–21. 15531878 10.1038/nature03104

[12] Bi F, Huang C, Tong J, Qiu G, Huang B, Wu Q, et al. Reactive astrocytes secrete lcn2 to promote neuron death. Proc Natl Acad Sci U S A. 2013;110(10):4069–74. 23431168 10.1073/pnas.1218497110 PMC3593910

[13] Jin M, Jang E, Suk K. Lipocalin-2 acts as a Neuroinflammatogen in Lipopolysaccharide-injected mice. Exp Neurobiol. 2014;23(2):155–62. 24963280 10.5607/en.2014.23.2.155 PMC4065829

[14] Jung BK, Park Y, Yoon B, Bae JS, Han SW, Heo JE, et al. Reduced secretion of LCN2 (lipocalin 2) from reactive astrocytes through autophagic and proteasomal regulation alleviates inflammatory stress and neuronal damage. Autophagy. 2023;19(8):2296–317. 36781380 10.1080/15548627.2023.2180202 PMC10351455

[15] Field-Smith A, Morgan GJ, Davies FE. Bortezomib (Velcadetrade Mark) in the treatment of multiple myeloma. Ther Clin Risk Manag. 2006;2(3):271–9. 18360602 10.2147/tcrm.2006.2.3.271 PMC1936263

