# Supplementary material for: Proteasome inhibition suppresses the induction of lipocalin-2 upon systemic lipopolysaccharide challenge in mice
Source: Mol Brain. 2024 Oct 3;17:73. doi: 10.1186/s13041-024-01147-w (PMC11451108; doi:10.1186/s13041-024-01147-w)
Supplement: Supplementary file 1 — Supplementary Material 1 [file 13041_2024_1147_MOESM1_ESM.docx]

**Supplementary information**

**Proteasome inhibition suppresses the induction of lipocalin-2 upon systemic lipopolysaccharide challenge in mice**

Jin-Sil Bae, Ji-Eun Heo, Kwon-Yul Ryu^*^

*Department of Life Science, University of Seoul, Seoul 02504, Republic of Korea*

*Corresponding author: Kwon-Yul Ryu, Department of Life Science, University of Seoul, 163 Seoulsiripdae-ro, Dongdaemun-gu, Seoul 02504, Republic of Korea.

*E-mail address:* kyryu@uos.ac.kr (K.-Y. Ryu)

**Materials and methods**

**Mouse experiments**

CD-1 (ICR) mice were purchased from Raon Bio (Yongin, Korea) and housed in plastic cages with *ad libitum* access to food and water under a 12-h light/dark cycle. All animal procedures were approved by the University of Seoul Institutional Animal Care and Use Committee (approval no. UOS-IACUC-2020-03-A, UOS IACUC-2021-01-TA). All mouse experiments were carried out according to the relevant guidelines and regulations approved by the UOS IACUC.

**Immunoblotting analysis**

Tissue lysates were prepared from mice treated with LPS (L2630; Sigma-Aldrich, St. Louis, MO, USA) and/or BTZ (S1013; Selleckchem, Houston, TX, USA) using TBSE buffer (50 mM Tris-HCl, pH 7.2, 150 mM NaCl, 1 mM EDTA with protease inhibitors [1 μg/mL aprotinin, 1 μg/mL leupeptin, 1 mg/mL PMSF]). Following a 30 min incubation on ice, the lysates were centrifuged at 13,000 × g for 15 min at 4 °C, and the supernatant was collected. We determined the protein concentration using the Pierce^TM^ BCA assay kit (Thermo Fisher Scientific, Waltham, MA, USA). Proteins (15–30 μg) were subjected to SDS polyacrylamide gel (10%) electrophoresis and then transferred onto polyvinylidene fluoride membranes. Membranes were blocked with 5% skim milk in Tris-buffered saline (TBS) containing 0.05% Tween-20 (TBST) for 1 h at room temperature and incubated with primary antibodies in 5% skim milk/TBST at 4 °C overnight. Following primary antibody incubation, the membranes were washed with TBST and then incubated with secondary antibodies (horseradish peroxidase [HRP]-conjugated goat anti-mouse IgG or rabbit IgG) in 5% skim milk/TBST for 1 h at room temperature. Chemiluminescent signals were detected using an enhanced chemiluminescence solution (WSE-7120L; ATTO, Tokyo, Japan), and images were captured with a ChemiDoc system (Bio-Rad, Hercules, CA, USA). The antibodies used in this study are as follows: anti-Lcn2 (PA5-79590; rabbit polyclonal, 1:1,000; Invitrogen, Carlsbad, CA, USA); anti-β-actin (SC-47778; mouse monoclonal, 1:1,000; Santa Cruz Biotechnology, Dallas, TX, USA); and HRP-conjugated goat anti-mouse or anti-rabbit IgG (ADI-SAB-100-J or ADI-SAB-300-J; goat polyclonal, 1:10,000; Enzo Life Sciences, Farmingdale, NY, USA).

**Quantitative reverse transcription polymerase chain reaction (qRT-PCR) analysis**

After treatment with LPS and/or BTZ, total RNA was isolated from tissues using TRI Reagent (Molecular Research Center, Cincinnati, OH, USA) according to the manufacturer’s protocol and resuspended in RNase-free double-distilled H_2_O. We determined RNA concentration using a NanoDrop One^TM^ instrument (Thermo Fisher Scientific). To remove any genomic DNA contamination, 1 μg of RNA was incubated with DNase I (Invitrogen) for 15 min at room temperature, followed by inactivation of DNase I. The RNA samples were used as templates for reverse transcription using an oligo(dT) primer (18-mer) and SuperiorScript II reverse transcriptase (Enzynomics, Daejeon, Korea), according to the manufacturer’s protocol. cDNA samples obtained from the mRNA were used as templates for qRT-PCR using 2× SYBR Master Mix (Enzynomics), forward and reverse primers, and an iCycler system (IQ5; Bio-Rad). The mRNA expression levels of *Lcn2*, *Tnfa*, *Gfap*, *Iba1*, and other astrocyte markers were normalized to the levels of glyceraldehyde 3-phosphate dehydrogenase (*Gapdh*). The major primers used in this study were as follows: *Lcn2*-F, 5′-CTG AAT GGG TGG TGA GTG TG-3′; *Lcn2*-R, 5′-GCT CTC TGG CAA CAG GAA AG-3′; *Tnfa*-F, 5′-TCT CAT CAG TTC TAT GGC CC-3′; *Tnfa*-R, 5′-GGG AGT AGA CAA GGT ACA AC-3′; *Gfap*-F, 5′-CGA GTC CCT AGA GCG GCA AAT G-3′; *Gfap*-R, 5′-GTA GGT GGC GAT CTC GAT GTC-3′; *Iba1*-F, 5′- GGA CAG ACT GCC AGC CTA AG-3′; *Iba1*-R, 5′- GAC GGC AGA TCC TCA TCA TTG-3′; *Gapdh*-F, 5′-GGC ATT GCT CTC AAT GAC AA-3′; and *Gapdh*-R, 5′-CTT GCT CAG TGT CCT TGC TG-3′. The primer sequences used for the other astrocyte markers are provided in Table 1.

**Statistical analysis**

To compare data between two groups, we used two-tailed unpaired Student’s t-tests in this study. We considered differences to be statistically significant at *p* < 0.05.

Table 1 Summary of the primer sequences used for pan, A1, and A2 astrocyte markers.

| **Genes** |  | **Sequences (5’-3’)** |
| --- | --- | --- |
| *Serpina3n* | F | CCT GGA GGA TGT CCT TTC AA |
|  | R | TTA TCA GGA AAG GCC GAT TG |
| *Osmr* | F | GTG AAG GAC CCA AAG CAT GT |
|  | R | GCC TAA TAC CTG GTG CGT GT |
| *H2-T23* | F | GGA CCG CGA ATG ACA TAG C |
|  | R | GCA CCT CAG GGT GAC TTC AT |
| *Serping1* | F | ACA GCC CCC TCT GAA TTC TT |
|  | R | GGA TGC TCT CCA AGT TGC TC |
| *S100a10* | F | CCT CTG GCT GTG GAC AAA AT |
|  | R | CTG CTC ACA AGA AGC AGT GG |
| *Clcf1* | F | CTT CAA TCC TCC TCG ACT GG |
|  | R | TAC GTC GGA GTT CAG CTG TG |
|  |  |  |

**Supplementary Figures**

**
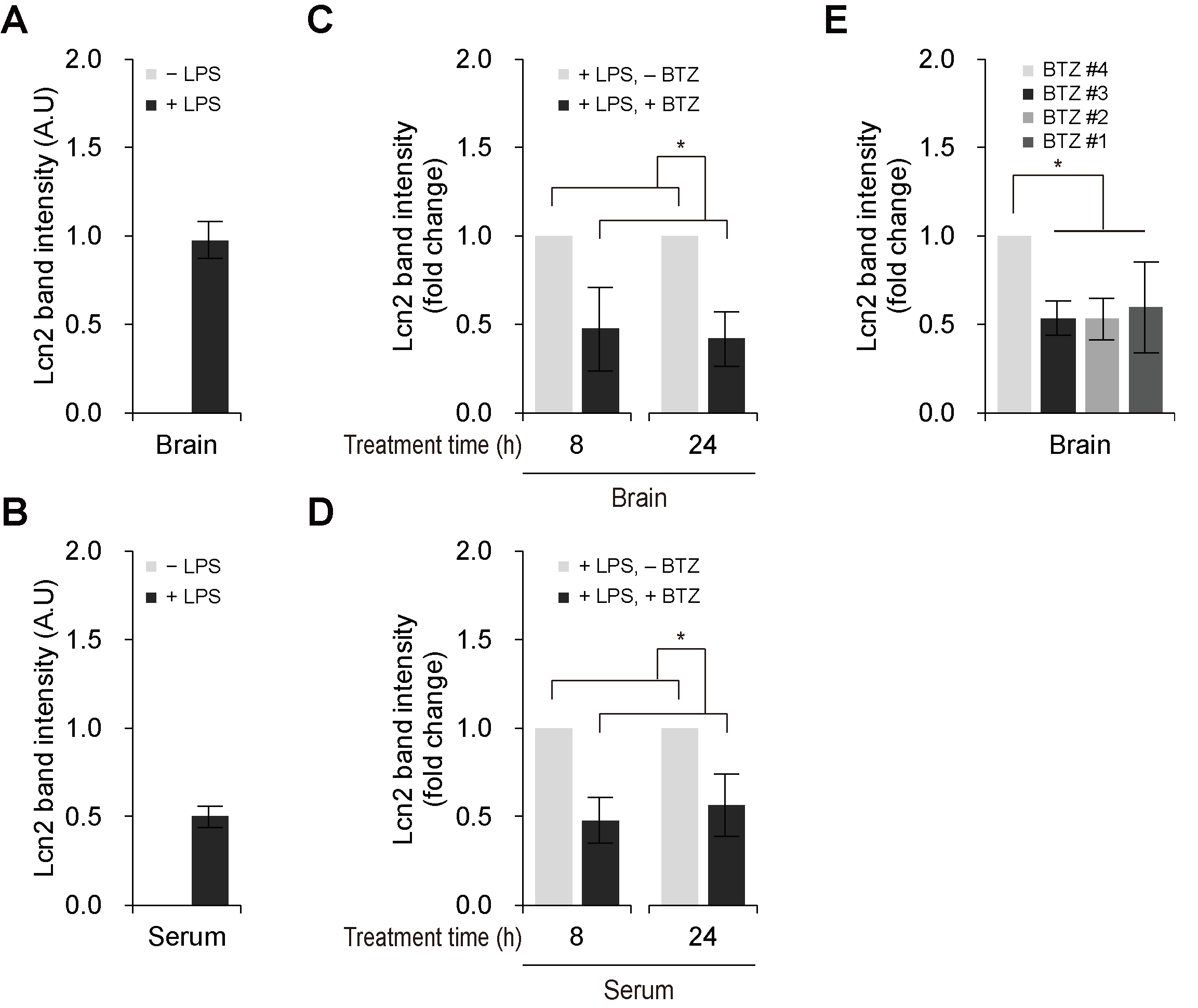
**

**Fig. S1** Quantification of immunoblot bands. (A, B) Band intensities of Lcn2 in Fig. 1A and Fig. 1B were normalized to the loading control β-actin or Ponceau staining and expressed as arbitrary units (A.U). (C, D) Band intensities of Lcn2 in Fig. 1C and Fig. 1D (+LPS, −/+BTZ, 8 or 24 h) were normalized to the loading control β-actin or Coomassie blue staining and expressed as the fold change relative to the control (+LPS, −BTZ, 8 or 24 h; n=2). (E) Band intensities of Lcn2 in Fig. 1F were normalized to the loading control β-actin and expressed as the fold change relative to the control (lane 4: BTZ #4; n=2). 'BTZ #1', 'BTZ #2', 'BTZ #3', and 'BTZ #4' refer to BTZ treatment for the last 18, 12, 6, or 0 h, respectively, during the 24-h LPS treatment. All band intensities were quantified using ImageJ software (version 1.54d). Data are expressed as means ± SEM. **p* < 0.05.


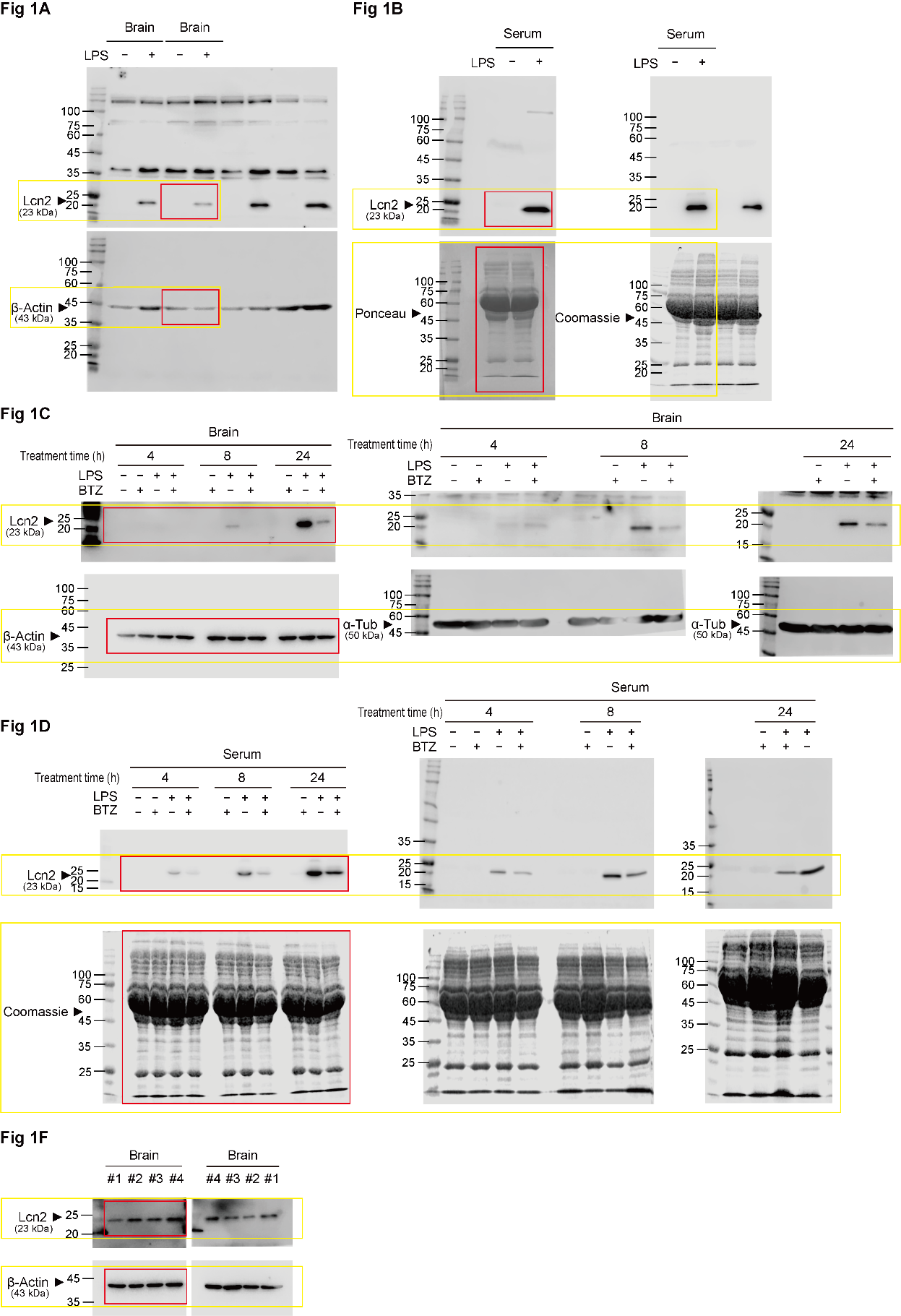


**Fig. S2** Uncropped immunoblot images.
